# To Do or Not to Do: Dopamine, Affordability and the Economics of Opportunity

**DOI:** 10.3389/fnint.2018.00006

**Published:** 2018-02-13

**Authors:** Jeff A. Beeler, Devry Mourra

**Affiliations:** ^1^Department of Psychology, Queens College, City University of New York, New York, NY, United States; ^2^CUNY Neuroscience Consortium, The Graduate Center, City University of New York, New York, NY, United States

**Keywords:** neuroeconomics, energy management, dopamine, basal ganglia, affordability, dopamine D2 receptor, striatum, behavioral thrift

## Abstract

Five years ago, we introduced the thrift hypothesis of dopamine (DA), suggesting that the primary role of DA in adaptive behavior is regulating behavioral energy expenditure to match the prevailing economic conditions of the environment. Here we elaborate that hypothesis with several new ideas. First, we introduce the concept of affordability, suggesting that costs must necessarily be evaluated with respect to the availability of resources to the organism, which computes a value not only for the potential reward opportunity, but also the value of resources expended. Placing both costs and benefits within the context of the larger economy in which the animal is functioning requires consideration of the different timescales against which to compute resource availability, or average reward rate. Appropriate windows of computation for tracking resources requires corresponding neural substrates that operate on these different timescales. In discussing temporal patterns of DA signaling, we focus on a neglected form of DA plasticity and adaptation, changes in the physical substrate of the DA system itself, such as up- and down-regulation of receptors or release probability. We argue that changes in the DA substrate itself fundamentally alter its computational function, which we propose mediates adaptations to longer temporal horizons and economic conditions. In developing our hypothesis, we focus on DA D2 receptors (D2R), arguing that D2R implements a form of “cost control” in response to the environmental economy, serving as the “brain’s comptroller”. We propose that the balance between the direct and indirect pathway, regulated by relative expression of D1 and D2 DA receptors, implements affordability. Finally, as we review data, we discuss limitations in current approaches that impede fully investigating the proposed hypothesis and highlight alternative, more semi-naturalistic strategies more conducive to neuroeconomic investigations on the role of DA in adaptive behavior.

## Introduction

Though studied for over half a century, the dopamine (DA) system continues to pose unanswered questions and inspire controversy. Newer methods have yielded advances in our understanding but at the same time opened up new questions. For many years, debate focused on whether DA modulated appetitive behavior through effects on motivation or learning (Wise, 2004; Salamone et al., 2005; Berridge, 2007), both anchored in the history of psychological theory. Recent years have seen growing interest in a neuroeconomic perspective on DA (Glimcher et al., 2005; Phillips et al., 2007; Kable and Glimcher, 2009; Sharp et al., 2012; Schultz et al., 2015). Traditional approaches draw upon psychological and physiological concepts about motivation and regulation, such as drive, reinforcement, and homeostasis as underlying determinants of behavior—asking how DA mediates these mechanisms. In keeping with Marr’s levels of analysis (Marr, 1982), a neuroeconomic perspective begins by defining the computational problem; broadly, adaptation and survival poses an essentially economic question: how to optimally deploy organismal resources to obtain maximal benefit in a given environment, yielding the greatest probability of survival (Glimcher, 2003). Within neuroeconomics, DA has figured prominently as a key neural substrate for tracking the value of stimuli and actions and modulating decision-making accordingly.

This shift in conceptual framework is illustrated in a recent study by Berke and colleagues (Hamid et al., 2016) where using both microdialysis and fast-scan cyclic voltammetry, they carefully measured DA signaling in rats during a probabilistic selection task. Their results effectively integrate previous competing views on DA. With regards to the debate on whether DA acts primarily by: (i) enhancing motivation and energizing behavior towards appetitive goals (Robbins and Everitt, 1992; Berridge et al., 2009; Salamone and Correa, 2012); or (ii) providing a teaching signal, modifying behavior through reinforcement learning (Schultz et al., 1997; Montague et al., 2004; Wise, 2006), they observe DA signals consistent with *both*. That is, increases in DA preceded increased motivated behavior, but prior DA activity affected subsequent behavior as well. What is crucial is the way in which they formulate the integration of these previously competing conceptualizations of DA function: DA signals the *value of work*. This three word integration represents a neuroeconomic conceptualization where the integration of various aspects of DA subserve a crucial organismal computational (and economic) challenge: deciding how to allocate resources to maximize return.

DA, often referred to as the “reward neurotransmitter”, is widely associated with regulating appetitive motivation. However, DA also regulates behavioral activation in a more generalized way, increasing or decreasing behavioral energy expenditure—activity—independent of pursuit of appetitive goals (for review, Beeler et al., 2012c). Salamone et al. (2007) have long argued that DA regulates an animal’s willingness to work, effectively energizing behavior to allow animals to overcome costs associated with pursuing goals. Following a set of studies conducted in homecage operant boxes with hyperdopaminergic mice (Beeler et al., 2010, 2012b,d), in which the results did not fit neatly into any reward-oriented theory of DA, and building on Salamone’s work, we proposed the thrift hypothesis of DA (Beeler, 2012; Beeler et al., 2012c). In brief, we argued that the primary function of DA was to regulate behavioral energy expenditure, which was accomplished along two dimensions or axes: (1) regulating a generalized willingness to expend energy along a continuum from energy conservation/storage to liberal energy expenditure/utilization; and (2) an additional dimension in which DA regulates how “careful” an organism is in its energy expenditure, which we conceptualized as regulating how tightly behavioral choice is coupled to prior learning about value (Beeler et al., 2010; Kayser et al., 2015), commonly known as the explore-exploit balance (Sutton and Barto, 1998; Daw et al., 2006). This latter captures the “reward” aspect of DA by determining the extent to which energy would be directed toward previously rewarding activities. Our premise was that DA adapted to a rich or poor environmental economy by either up- or down-regulating, respectively, which in turn favored energy expenditure and exploration (maximal utilization of resources and opportunities) or energy conservation and maximal exploitation of prior learning (effectively also maximal utilization of *scarce* resources).

In this review and hypothesis article, we elaborate the rudimentary thrift hypothesis focusing on the neglected question of how the *availability of resources*—both internal and external—are factored in to DA-mediated computations and signaling about value, and corresponding decisions to expend energy or not: *to do or not to do*. Our aim is to develop a basic evolutionary perspective on DA: as animals cannot control resource availability, all adaptation and survival necessarily devolves to the problem of optimally expending energy within the constraints of the economic environment in which the animal finds itself. That is, the animal cannot control its environment, but it can control its choices within that environment, specifically how it utilizes the energy it has available. A central concern here is the last part: how does an organism, and putatively the DA system, assess resource availability and incorporate that into computations signaling “the value of work” that regulate behavioral choice and energy expenditure.

## Reward, Value and the Neglected Question of Affordability

Within the “motivational perspective” of DA, debate has continued as to whether, in cost-benefit computations, DA is modulating cost or benefit. Berridge and Robinson (1998), in their incentive-salience theory, suggest that DA enhances the incentive associated with stimuli, thus propelling expenditure of energy toward those stimuli, effectively driving behavior by increasing expected “benefit”. Salamone et al. (2007), in contrast, have argued that DA enhances motivation by increasing an animal’s willingness to work toward some goal, driving behavior by decreasing cost sensitivity. It is practically difficult to discern the difference between these: if expected benefit is increased, this will increase the costs an animal is willing to incur, leading to increased effort. Conversely, if sensitivity to costs is reduced, this effectively increases benefit *relative* to costs—and again, the animal works harder. While the recent work of Hamid et al. (2016) offers an elegant integration of these two ideas in their formulation that DA signals “the value of work”, this formulation provokes further questions.

Hamid et al. (2016) suggest that DA signals instantaneous value, which at target regions could *both* increase the incentive associated with relevant stimuli (increase expected benefit) and energize responses (decrease cost sensitivity). That is, DA instructs the animal both how valuable the stimuli/reward are and how much effort should be expended. In a sense, this begs the question: how are costs and benefits weighed and factored in to generate an instantaneous value signal that both increases incentive and decreases cost-sensitivity. Simplistically, acceptable costs and benefit should mirror each other such that the greater the benefit, the greater the willingness to incur costs. However, the factors that determine cost and benefit are different. Benefit is computed based on utility: need or desire. Cost, however, is contingent upon available resources, i.e., *ability to pay*. Consequently, the willingness to incur costs does not necessarily scale with perceived benefit. Intuitively, in our daily lives what *primarily* constrains expenditures is not the perceived value of goods, but the resources we have to exchange for those goods; that is, rather than scaling acceptable costs to benefit, we typically have to scale perceived benefits to *affordable* costs. Affordability weighs the value (utility, benefit) of the potential reward against the ***value of the resources that must be given up*** to obtain that reward (costs), the latter value determined by the animal’s available resources or *wealth*. For example, for an animal with energetic wealth (plentiful internal stores of energy, rich energy environment), the value of energy expended in lever pressing might be very low as energy is in plentiful supply, and thus lever pressing costs may factor very little compared to the expected benefit of the reward. In contrast, for an animal in energy deficit in a scarce environment, energy expended in lever pressing may be very valuable, and thus weigh against expected benefit of reward much more greatly, depressing the net value in cost-benefit calculations. Put another way, before an animal can determine how many lever presses a given reward may be worth, the animal has to place a value on the lever presses. By analogy, before I can determine if a purchase is worth $10.00, I have to determine the worth of $10.00, which will differ greatly depending upon my general wealth, whether I am a millionaire or a poor graduate student. If DA is providing an instantaneous value signal, does this signal incorporate affordability?

The extent to which a putative DA value signal is modulated by resource availability, i.e., affordability determined by the animal’s “economic circumstances”, has been little investigated. Both Salamone’s work and our own suggest that DA mediated changes in behavioral energy expenditure are not limited to appetitive pursuit; generalized, non-appetitive behavior is altered (e.g., open field, wheel-running) and even in appetitive activities, increased energy expenditure does not necessarily lead to increased consumption; for example, hyperdopaminergic animals will work harder for the *same amount* of food (Beeler et al., 2010, 2012b). If energy is taken as a form of currency, these data suggest that increased DA signals energy wealth and promotes profligate rather than penurious expenditure (Beeler et al., 2012c). Returning to Hamid et al. (2016) notion of an instantaneous value signal, we suggest that affordability (i.e., *value of resources expended*) can be implemented in two ways: (1) by directly modulating the DA value signal itself; and/or (2) by altering the response to the DA value signal at targets regions, potentially modulating incentive and cost-sensitivity independently. We will argue for both and suggest that DA D2 receptor (D2R) plays a central role in incorporating affordability into DA mediated value signaling.

### Timescales: Windows of Computation

By “timescale” we mean both the period of time against which computations of available resources are calculated, both internal (organismal) and external (environmental), as well as the temporal scale(s) in which DA signaling is modulated. In foraging theory, a cardinal challenge facing animals foraging for food is known as the patch-leaving problem (Stephens and Krebs, 1986). In brief, if an animal is in a patch of food that is being depleted, such that obtaining additional food requires more time and/or effort, at what point should the animal leave the patch and seek a new food source? This pits the increasing costs associated with the current patch against the costs (time, effort, risk) of finding a new patch. The classical solution to this, known as the marginal value theorem, suggest that the optimal strategy is to leave the patch when the local rate of return drops below the average rate of return over time in the environment (Charnov, 1976; Constantino and Daw, 2015). This leaves open the question, though, of what period of time should be used to compute the average rate of return? Average rate of return for *that day* of foraging? The last few days? During this *season* (months)? Some lifetime cumulative average? A case could be made for all of these: rates of return might vary daily based on factors such as weather (windy day blowing acorns off trees or a rainy day bringing worms out of the ground, for example), shorter periods of days/weeks may vary based on growth cycles of particular plants. Clearly seasonal variation in availability of resources should be taken into account—winter foraging using a summer average rate of return could be catastrophic. Longer periods, such as might be associated with extended droughts or other fluctuations would be appropriate to take into consideration, and finally, lifetime rate of return may, importantly, reflect the general harshness of the organism’s environment, such as the difference between a desert and a farm mouse. These different possible timescales for evaluation of resource availability generate both a theoretical question—what would be the *optimal* choice of timescale(s)—as well as a practical, empirical question: do animals, including humans, track average value on different timescales, and if so, how is this computed and signaled and how does it contribute to decision-making?

The question of the appropriate temporal horizon against which to calculate averages has a corollary question: what is the optimal *learning rate*. That is, how readily should new information update prior knowledge? Mathematically, learning rate effectively determines the period of information taken into account. A high learning rate means that new information quickly outweighs prior information, favoring recency and a shorter window of averaging. Conversely, a low learning rate gives little weight to new information, favoring cumulative information over a longer period of time. Thus, in addition to the question of what are appropriate periods against which to evaluate resource availability, there is the intimately related question of what are appropriate, or optimal, learning rates by which new information should be incorporated and weighed against prior information. Optimal learning rates are contingent upon environmental conditions. In a highly variable, “noisy” environment, responding to rapid fluctuations rather than broader trends might result in suboptimal decision-making. Conversely, in a more stable environment, failure to respond to a significant change rapidly may result in lost opportunity and reduced adaptation.

Though the question of what time scales are relevant and appropriate for consideration is more obvious in the case of external, environmental resources, the same question also applies to internal, organismal resources. For brevity, we highlight the temporal difference in insulin and leptin, signaling immediate energy resources and long-term energy stores, respectively. The same issues pertain: the degree to which a transient decrease in energy (i.e., reduced blood glucose) may increase the cost an animal is willing to expend to obtain food may vary depending upon long-term stores, or reserves, of energy.

An important aspect of tracking resource availability is that, to an extent, *experienced* resource availability is dependent upon the animal’s choices. For example, an animal that persists longer than optimal in a depleting patch will, over time, depress their average rate of return (e.g., Wikenheiser et al., 2013). Insofar as this average rate of return contributes to decision-making, the possibility exists of entering a self-imposed, vicious cycle of diminishing returns. This mismatch between behavior and actual conditions can be observed with regards to internal resources as well, as observed in obesity where an animal with “excess” reserves may, nonetheless, favor persistent conservation and storage. In effect, an organism’s computations and subsequent choices can create a perceived or experienced environment inconsistent with the actual environment in which the animal is functioning.

In asking how resource availability might be tracked, computed and contribute to decision-making—incorporating “affordability”—the question of time scale, or temporal windows for tracking and computation, plays a critical role in defining the context of decision-making. As we hypothesize that DA signaling is modulated by resource availability, signaling affordability as well as value, we turn next to the question of temporal patterning of DA signals.

### Timescales: Temporal Characteristics of DA Signaling

Based on early electrophysiological observations, DA cell activity has long been characterized as having two modes of firing (Grace and Bunney, 1984a,b; Hyland et al., 2002). Tonic refers to on-going, low frequency (~4 Hz) irregular firing intermittently interrupted with short, high frequency bursts of actions potentials, or phasic activity. Tonic activity is believed to maintain extracellular DA at relatively stable concentrations as the primary mode of clearance at low release rates is diffusion (Venton et al., 2003; Cragg and Rice, 2004; Arbuthnott and Wickens, 2007). Phasic activity, in contrast, generates transient elevations of DA at a subsecond timescale that can be correlated to events and stimuli occurring in the animal’s environment, suggesting phasic signaling is responsive to on-going sensory experience (Schultz et al., 1993; Carelli and Wightman, 2004; Roitman et al., 2004; Stuber et al., 2005; Tobler et al., 2005; Cheer et al., 2007; Day et al., 2007; Schultz, 2007a; Bromberg-Martin and Hikosaka, 2009; Bromberg-Martin et al., 2010; Cameron et al., 2014; Hart et al., 2014, 2015; Kishida et al., 2016). Schultz (2007b) proposed that tonic DA modulates motivational processes while phasic DA, temporally synchronized with sensory events, mediates reinforcement learning. Niv et al. (2007) proposed that tonic DA tracks average reward rate over time, such that greater average reward increases tonic DA. Increased tonic DA, in turn, energizes behavior in response to overall greater reward availability, minimizing the opportunity costs associated with failure to harvest available reward, memorably expressed as “the cost of sloth”.

The notion that DA signals through two distinct modes, tonic and phasic, is not without critics. Recent studies have observed a generalized increase in DA—not associated with temporally discrete stimuli such as cues—within the context of a task where a hungry animal has an opportunity to earn food, an effect that involves learning about the potential value associated with the task-environment (context) as this effect increases across training trials (Howe et al., 2013; Hamid et al., 2016). While it might be tempting to think of this generalized, task-related increase in DA as “tonic”, this only opens the door to the sort of questions asked above about the timescales on which “tonic” DA operates, as well as how tonic activity is regulated in association with on-going sensory experience. The generalized increase in DA signaling observed in the task context by both Howe et al. (2013) and Hamid et al. (2016) presumably reflects learning about contextual stimuli that predict reward availability similar to discrete cues but with less temporal specificity. Hamid et al. (2016) argue against the idea of distinct tonic and phasic DA signals and propose instead that at any given moment DA provides an instantaneous value signal. We agree and would argue that the moment-to-moment readout of DA activity arises as a compound signal integrating value information on multiple timescales. That is, the presence of contextual stimuli associated with increased probability of reward (e.g., task environment) is compounded with more temporally resolved stimuli (e.g., discrete cues) to generate fluctuations in instantaneous probability of reward.

Consequently, we would modify the hypothesis of Niv et al. (2007), retaining the notion that average rate of reward factors into DA signaling, but, like Hamid et al. (2016), rejecting the separation of DA activity into distinct tonic and phasic activity. Instead, opportunity for value/reward can be computed on multiple timescales—from seconds, such as the appearance of a cue-light indicating reward availability imminently, to minutes to hours, such as a constellation of stimuli indicating a context (task environment) associated with greater reward availability. This reformulation leaves open the question of how other timescales for computing the average reward rate may contribute to the observed patterns of DA signaling. That is, to what extent is task-associated increase observed in Hamid and Howe contingent on even broader timescales, such as the condition of extreme scarcity (i.e., 85% body weight food restriction) during the other 23 h of the day?

**Figure 1:**
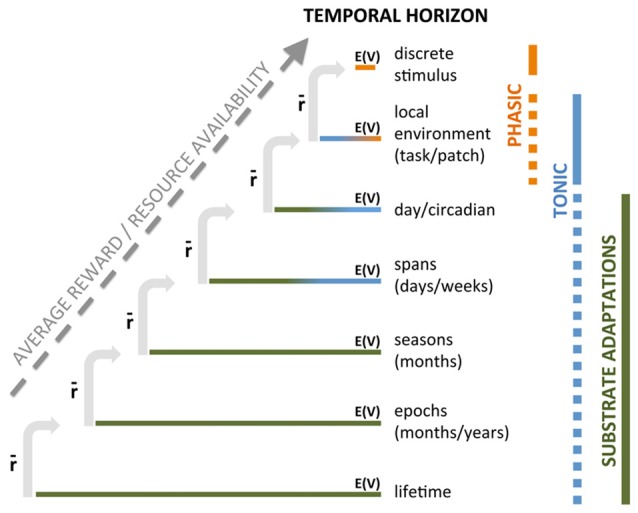
Nested temporal horizons for computing and signaling average rewardand opportunity. Each temporal period is associated with availability of reward opportunity, E(V), that should presumably motivate appropriate appetitive behavior in response. However, the value of that opportunity is contingent upon the average reward in the broader, containing temporal frame, denoted by $\bar{\text{r}}$. Average reward is presented as a progressive set of nested temporally defined contexts that determines the value of opportunity and resources expended in shorter periods of time. The extent to which DA transmits opportunity, E(V) or average reward, $\bar{\text{r}}$, by “phasic” or “tonic” DA cell activity or by alterations in the DA substrate itself is tentatively indicated by vertical bars on the right.

We propose that different timescales operate in a nested fashion to produce a compound instantaneous DA signal: expected value (opportunity) at a given timescale is contingent upon the average rate of reward in the broader, enclosing timescale (Figure 1). Thus, we would argue that the context-associated increase in DA across a task is predicated on the larger timescale of food restriction (FR). Similarly, the expected value of a cue in a task is predicated on the richness of the task environment; for example, we would predict that greater inter-trial intervals (less overall reward in task) would decrease the across-task increase in DA but enhance the cue-specific DA response because the opportunity indicated by the discrete cue is more valuable when the task has lower overall reward availability. Conversely, increasing reward opportunities in a session would increase across-task DA while likely diminishing cue-dependent DA responses as each individual cue is proportionally less valuable in a richer task environment. In short, value computed at one timescale is always contingent upon rate of reward, or available resources, at a broader timescale.

This notion of nested timescales of value makes sense in another way. Niv et al. (2007) propose that increases in so-called tonic DA increase vigor, but this would only be useful at those moments when an opportunity to respond (e.g., press lever) is available. What function would increased DA do *between* response opportunities? We suggest DA signaling arising from slower timescales (e.g., task) energizes behavior without a specific target to facilitate exploration of the environment, scaled appropriately to the richness of the environment, while a surge in DA at a shorter timescale would refocus the animal on the more discrete predictive cue, reorienting behavior to the cue/task, as suggested by Nicola’s flexible approach hypothesis (Nicola, 2010). Notably, in a very rich task environment, animals may miss more opportunities for reward, which could be explained by increased DA signaling at the task timescale, promoting exploration of the environment concomitant with decrease signaling in response to discrete cues (because they are less valuable in an environment where they occur more frequently), diminishing the strength of orienting to cues within the task. While this discussion centers on timecourse(s) of DA signaling, the crucial observation for the hypothesis being developed here is that these nested timescales of average reward (broader timescale) and expected value (narrower timescale) provide a mechanism by which value computations at every temporal resolution can be computed in the context of a more general resource availability, or average rate of reward.

### Computation vs. Computational Adaptation

Implicit in the question of “what does DA encode” is the idea that DA cells integrate multiple inputs, perform some transformation (computation) and signal value to energize behavior, in which the output signal is a function of the input, i.e., DA signal = *f*(*input*_1−*n*_). This takes for granted that the cellular machinery performing this transformation/computation is constant, reliably implementing some optimizing algorithm to compute value. We know, however, that different functional components of the DA system are not constant and are subject to regulated up- and down-modulation, including expression/function of: (i) the DA transporter (DAT) regulating reuptake and the duration of signaling arising from (burst) release events (Kristensen et al., 2011); (ii) tyrosine hydroxylase, regulating the rate of synthesis of DA (Kaushik et al., 2007; Daubner et al., 2011); (iii) vmat2, regulating the packaging of DA into vesicles (Pifl et al., 2014); (iv) readily releasable pools (Turner, 2004), regulating the ability of DA terminals to sustain release in bursts; and (v) receptors (e.g., Knab and Lightfoot, 2010; Kenny et al., 2013; Petzinger et al., 2015; Friend et al., 2016), whose up- and down-regulation can alter the cellular transduction of any given DA signal. Though this regulated, functional plasticity in the DA system has been extensively studied in pathological conditions, there has been no systematic investigation of the adaptive purpose, if any, of this capacity for regulation of the DA system under non-pathological conditions. Nonetheless, if the characteristics of the substrate upon which some neuroeconomic computation is carried out are changed, it seems likely that the result of the computation would also change. That is, the function that defines the relationship between input to midbrain DA and its subsequent output and downstream effects is dependent upon the regulated properties of the various components of DA substrate mediating this function.

**Figure 2:**
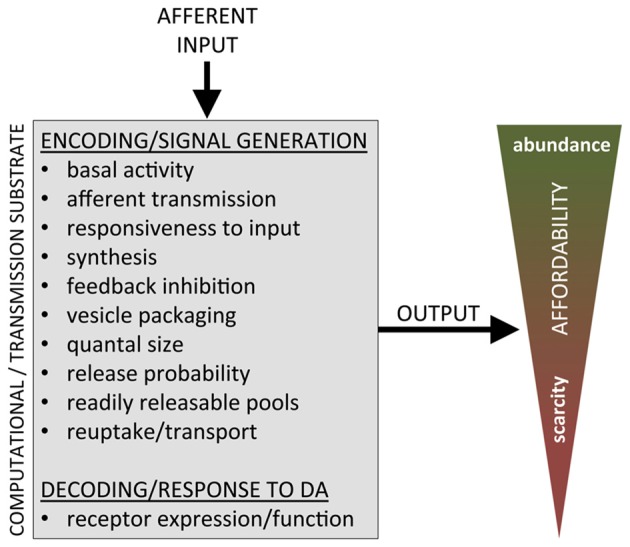
Functional aspects of the DA system substrate available for regulation in putatively implementing affordability to adapt to economic conditions.

Our hypothesis is that the tracking of resources, both internal and external, and the subsequent incorporation of this information into neuroeconomic decision-making as *affordability*, is implemented at a level of adaptation that functionally alters the computational substrate itself, the DA system. When the computational substrate is altered, so is the computation, such that any abstract function that relates DA system input to output cannot be taken for granted but has to qualify the characteristics of the DA system that is performing that function: DA signal = *f*(K, *input*_1−*n*_), where K is a vector characterizing the state of different functional aspects of the DA system, such as release probability, reuptake, receptor density and so on. Simply put, we propose: (i) the DA substrate undergoes (genetic and other) regulation to adapt the computational substrate—and the computations—that determine the *value of work*; (ii) in accordance to the prevailing economy in which the animal finds itself, determined by the general availability of resources, both internal and external; and (iii) effectively scaling the value of work—the value of the energy *expended*—to match available resources, i.e., incorporating *affordability* into value computations (Figure 2).

## Adapting to the Economic Climate: DA as Regulator of Investment Strategy

The idea that aspects of the DA system can change is not new, and has been examined in a variety of contexts, mostly pathological, including addiction (Lüscher and Malenka, 2011; Volkow and Morales, 2015), obesity (Baik, 2013; Kenny et al., 2013; Naef et al., 2015; Décarie-Spain et al., 2016) and Parkinson’s and Huntington’s disease (Dauer and Przedborski, 2003; Calabresi et al., 2009; Dawson et al., 2010; Chen et al., 2013; Surmeier et al., 2014; Bastide et al., 2015). In addition, it has been studied in combination with environmental factors, including diet (South and Huang, 2008; Morris et al., 2011; Speed et al., 2011; Cone et al., 2013; Adams et al., 2015; Hryhorczuk et al., 2016; Fordahl and Jones, 2017), FR (Zhen et al., 2006; Sevak et al., 2008; Carr et al., 2009; Branch et al., 2013; Stouffer et al., 2015; Fordahl and Jones, 2017), stress (Abercrombie et al., 1989; Cabib and Puglisi-Allegra, 1996, 2012; Gambarana et al., 1999; Mizoguchi et al., 2000) and exercise (Foley and Fleshner, 2008; Knab and Lightfoot, 2010; Vučković et al., 2010; Garland et al., 2011; Fisher et al., 2013; Petzinger et al., 2015). Similarly, the regulation of DA via internal signals such as insulin (Carvelli et al., 2002; Mebel et al., 2012; Labouèbe et al., 2013; Stouffer et al., 2015), leptin (Brunetti et al., 1999; Hommel et al., 2006; Perry et al., 2010; Billes et al., 2012), ghrelin (Jerlhag et al., 2007; Abizaid, 2009; van Zessen et al., 2012; Cone et al., 2014), glucocorticoids (Rougé-Pont et al., 1995, 1998; Piazza et al., 1996; Piazza and Le Moal, 1997; Van Craenenbroeck et al., 2005), GLP-1 (Dickson et al., 2012; Skibicka, 2013) and others has also been extensively studied. There is no doubt that the components of the DA system that comprise a computational substrate for economic decision-making are plastic, able to up- and down-regulate synthesis (Lindblom et al., 2006; Li et al., 2009), packaging and release (Turner, 2004; Lohr and Miller, 2014), reuptake (Jones et al., 2017) and receptor expression, trafficking and function (Tirotta et al., 2008; Knab and Lightfoot, 2010; Kenny et al., 2013; Petzinger et al., 2015; Friend et al., 2016).

However, a gap seems to exist between those that approach the question “what does DA encode”, using methods such as electrophysiology and fast-scan cyclic voltammetry (more recently photometry), often in conjunction with computational models, and those that study how the DA system itself can be altered by various environmental and organismal conditions. In particular, normative theoretical models (e.g., temporal difference learning) have not systematically or formally treated how changes in the DA substrate would alter this computational function. Conversely, those studying how a particular condition alters the DA system typically interpret findings in the context of the condition being studied—for example, how changes in DA facilitate obesity—without placing this in the larger context of the neuroeconomic role of DA in decision-making generally. This leaves a gulf between formal, normative computational accounts and physiologically oriented, systems-like engineering accounts of DA, a gap between coders and tinkerers. The result is a large lacuna: while we know the DA system can undergo adaptive changes, we study this primarily in pathological conditions and have little idea what the broader adaptive purpose of this evolved plasticity may be and how it may serve adaptive computational goals.

Approaching this lacuna requires a two-fold strategy: one, considering this substrate plasticity of DA not as arbitrary phenomena that arises under particular pathological conditions, but as an evolved mechanism serving a specific adaptive function, systematically investigating what factors, particularly environmental, determine the “set-point” of various aspects of the DA system, such as synthesis, receptor expression level and so on, and how that “set-point” alters decision-making and adaptive behavior. Two, in formal models of DA as a computational substrate, begin to ask how a change in one aspect of the DA system alters the resulting computations, matching elements of DA substrate to terms and parameters in models and adopting a normative perspective on substrate regulation of DA signaling.

## Controlling the Energy Budget: Striatum as Substrate Implementing Economy

**Figure 3:**
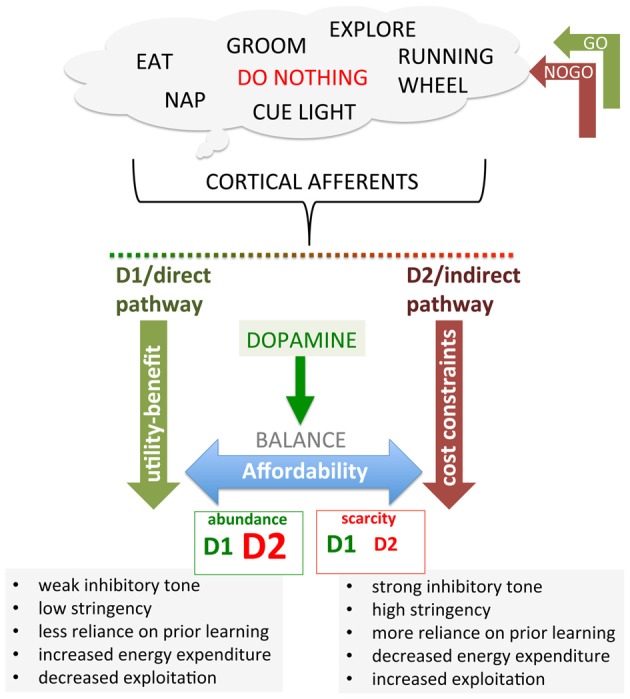
Recasting the direct (GO) and indirect (NOGO) corticostriatal pathways in terms of thrift and affordability. Cortical activities associated with different behavioral options send afferents to both the direct and indirect corticostriatal pathways. D1 and D2 expressed on direct and indirect pathway MSNs, respectively, both facilitate behavioral activation by facilitating and disinhibiting the associated cortical activity. Holding D1 constant for illustration, an increase in D2 expression confers greater disinhibition in response to DA, decreasing the threshold of facilitation necessary in the direct pathway to engage a behavior. Conversely, reduction in D2 confers a smaller disinhibition in response to DA, requiring greater facilitation from the direct pathway to overcome the inhibition. In the hypothesis proposed here, we suggest that the D1-expressing, striatonigral GO pathway effectively encodes utility/benefit while the D2-expressing, striatopallidal pathway implements cost constraints by up- and down-regulating D2 expression and function in response to general economic conditions, which effectively constrains facilitation by utility/benefit, implementing affordability.

In this section, we will focus on the basal ganglia, primarily the striatum, as a key substrate mediating DA-dependent neuroeconomic decision-making. Building on the distinction between value attributed to a potential reward (benefit) and the value assessed on the resources expended to obtain that reward (costs), we propose that striatal D1R signaling mediates computing the value of the reward pursued; that is, benefit or utility, while striatal D2R mediates computing the value of the resources expended. We propose that cost-benefit decision-making is modulated by the balance of between D1R and D2R signaling on the direct and indirect pathways (Figure 3), where D2R, adapting to available resources from abundance to scarcity, implements cost constraints that determine affordability.

### Dual Cortico-basal Ganglia-cortical Pathways: Accelerator and Brake for Energy Expenditure

The rich DA innervation of the striatum makes it an ideal candidate substrate for regulating thrift. Though the exact function of the basal ganglia remains controversial (Redgrave et al., 1999; Nambu, 2008; Desmurget and Turner, 2010; Shiflett and Balleine, 2011), cortico-basal ganglia-cortical re-entrant loops modulate and effectively filter cortical activity (Beeler, 2011; Beeler et al., 2013). As the primary input nucleus from the cortex to the basal ganglia, the striatum is a key substrate modulating this cortical filtering (Alexander and Crutcher, 1990; Parent and Hazrati, 1995; Lovinger, 2010). The dual pathway architecture in which the direct, or GO pathway facilitates cortical activity while the indirect, NOGO pathway inhibits it, provides a functional substrate for regulating behavioral thrift by providing, in effect, an accelerator and brake, respectively (Albin et al., 1989; DeLong, 1990; Gerfen, 1992; Mink, 1996; Frank, 2005; Kravitz et al., 2010; Beeler, 2011; Durieux et al., 2012; Freeze et al., 2013). The terms “accelerator” and “brake” are synonymous with “GO” and “NOGO” and not intended to imply a novel theory. Rather, because the GO/NOGO terminology is indelibly associated with the action selection hypothesis (Redgrave et al., 1999), where the GO pathway selects one action and the NOGO suppresses others (Mink, 1996), we shift to the “accelerator/brake” metaphor to dissociate the opposing facilitatory/inhibitory actions of the dual pathways from the selection of discrete actions and to emphasize a more *generalized* facilitation/inhibition of cortical activity. *Selective* facilitation/inhibition of particular cortical activity, as connoted by the terms GO/NOGO, depends upon corticostriatal plasticity and learning, which is also DA-dependent. Activation of D1R and D2R is required for long-term potentiation and long-term depression in the direct and indirect pathways, respectively, both of which facilitate selected cortical activity in future behavior (Calabresi et al., 2007; Surmeier et al., 2009; Lovinger, 2010; Lerner and Kreitzer, 2011). Thus, DA regulates both on-going, current behavior through modulation of MSN excitability as well as *future* behavior by modulating corticostriatal synaptic plasticity.

Through these dual pathways, DA regulates behavioral activation by gating the inhibitory tone on cortical activity. Increased DA can result in both stereotypic and disorganized behavior (Seeman and Kapur, 2000; Lewis et al., 2007; Tanimura et al., 2009; Langen et al., 2011; van Enkhuizen et al., 2014), increasing activity and energy expenditure, consistent with a released “brake” in combination with too much activation of the “accelerator”. In contrast, low DA results in too much brake and lack of accelerator, such as described in the classic model of motor deficits in Parkinson’s (Albin et al., 1989; DeLong, 1990; Frank, 2005; Kravitz et al., 2010). While the direct and indirect pathways could operate symmetrically, such that accelerator and brake mirror each other, there is no requirement for this to be so (Park et al., 2013; Tomer et al., 2013; Cazorla et al., 2014; Oldenburg and Sabatini, 2015; Nagano-Saito et al., 2017). In fact, it has frequently been observed that one population of receptors, either D1R or D2R, is up- or down-regulated while the other is not, indicating that the balance between these two circuits can be shifted. The relative expression of these two populations of striatal DA receptors will differentially alter the sensitivity of either the brake or accelerator to DA release. For example, if D2R were maximally expressed on the indirect pathway while D1R is greatly reduced, this would “open” the indirect pathway inhibitory gate on cortical activity without providing much selective facilitation in the direct pathway, generating increased but unfocused activity. Conversely, if D1R is maximally expressed but D2R is absent, DA would not release inhibition on cortical activity exerted by the indirect pathway, allowing only the strongest inputs through the direct pathway to facilitate cortical activity, presumably resulting in a paucity of behavior, permitting only the most highly motivated and reinforced behaviors to be expressed (Figure 3).

Similarly, selective facilitation/disinhibition arising from learning depends upon complementary corticostriatal plasticity in the two pathways, such that LTP in the direct pathway selectively facilitates cortical activity while LTD in the indirect pathway selectively releases the same activity from inhibition (Lovinger, 2010; Lerner and Kreitzer, 2011). But again nothing requires these complementary processes to be symmetrical or balanced. For example, a lack of D2R activation can impair LTD in the indirect pathway, even inverting it to LTP (Calabresi et al., 1997; Picconi et al., 2003; Kreitzer, 2005; Shen et al., 2008; Thiele et al., 2014). Independent regulation of these two populations of DA receptors, then, could also affect the relative balance of selective facilitation/inhibition through synaptic plasticity and learning (Wiecki et al., 2009; Wiecki and Frank, 2010; Beeler, 2011; Beeler et al., 2012a; Zhuang et al., 2013).

As noted, though we propose that striatal D1R and D2R mediates the modulation of utility and costs, respectively, in response to DA signals, from here we will focus only on D2R and the implementation of cost control.

### D2R: The Brain’s Comptroller?

D2R in the striatum has been associated with the regulation of motivated, appetitive behavior, including reinforcement learning and behavioral flexibility. D2R has also been implicated in generalized behavioral activation (Tataranni et al., 2001; Kravitz et al., 2010; Klinker et al., 2013; Beeler et al., 2016; Friend et al., 2016; Lemos et al., 2016; Thanos et al., 2016). Each could potentially explain the other; that is, blocking D2R could reduce motivation, effectively reducing activity or, conversely, a generalized restriction on activity and energy expenditure could diminish apparent motivation. The two alternative interpretations can be integrated by construing D2R expression as mediating a cost threshold—encoding the value of resources expended—that gates energy expenditure, affecting both willingness to expend energy in the pursuit of specific reward as well as generalized energy expenditure.

Because of its higher affinity for DA (Rice and Cragg, 2008), D2R activation in the striatum has been proposed to be largely saturated at tonic levels of extracellular DA. In contrast, D1R is not saturated and more responsive to phasic DA (Dreyer et al., 2010), suggesting that indirect pathway D2R regulates tonic inhibitory tone on cortical activity and, in turn, basal activity and behavioral energy expenditure. This is consistent with data that suggest D2R can regulate the functional balance in basal ganglia circuitry (Cazorla et al., 2014). A transient increase in DA, then, would act primarily through D1R to facilitate cortical activity in generating a response, but D2R in the indirect pathway determines the strength of the inhibitory tone that has to be overcome by D1R facilitation. Learning (LTD) in the indirect pathway can allow release of cortical inhibition from specific afferents, exempting selected cortical activity from basal inhibition, possibly by-passing “cost control”. However, alterations in D2R also modulate synaptic plasticity at corticostriatal synapses in the indirect pathway (Calabresi et al., 2007; Shen et al., 2008; Di Filippo et al., 2009; Lovinger, 2010; Lerner and Kreitzer, 2011; Thiele et al., 2014), setting a threshold for afferent activity required to induce LTD (Kheirbek et al., 2009; Wiecki et al., 2009; Augustin et al., 2014). By regulating the stringency of requirements for learning that affects future, selective release of inhibitory tone, D2R could provide a mechanism for regulating learning rate in response to DA signals.

Data are generally consistent with this proposal. Using pharmacology and lesion methods, Salamone and colleagues, as well as others, have repeatedly demonstrated that reduced DA, including reduced D2R activation specifically, diminishes an animal’s willingness to work for reward (Koch et al., 2000; Nowend et al., 2001; Baldo et al., 2002; Woolverton and Ranaldi, 2002; Salamone et al., 2007, 2009b; Pardo et al., 2012). As this is only observed at higher costs (ratio strain, Aberman and Salamone, 1999; Salamone et al., 2001), Salamone has consistently interpreted these data as reflecting increased sensitivity to costs (implying, in converse, that D2R activation decreases sensitivity to costs, Salamone et al., 2009a). Consistent with this, Soto et al. (2011) show that D2R KO mice show greater elasticity in their demand for food, such that as costs increase, they reduce their consumption to a greater extent than wild-type controls, despite presumably being more hungry, increasing the utility of food reward, as a consequence of consuming less. Blockade of D2R also reduces general behavioral activity, such as homecage or open-field activity, demonstrated most clearly with D2R knockout/knockdown mice that show reduced activity on various measures under various conditions (Tran et al., 2002; Klinker et al., 2013; Beeler et al., 2016; Friend et al., 2016; Thanos et al., 2016), data consistent with observation of a D2R reduced function polymorphism in humans that is associated with reduced activity (Tataranni et al., 2001).

In sum, D2R in the indirect pathway regulates the extent to which inhibition of cortical activity is released by DA, effectively setting a threshold gating DA-mediated behavioral activation. D1R in the direct pathway, responsive to transient increases in DA value signals, facilitates cortical activity and behavioral activation, implementing incentive associated with expected benefit or utility. This benefit-driven facilitation, however, must overcome D2R regulated indirect pathway inhibition. We propose that D2R up- and down-regulates in accordance with resource availability, particularly energetic wealth, providing a mechanism for implementing cost control; that is, by regulating the inhibitory threshold that must be overcome by direct pathway facilitation, D2R in the indirect pathway gates the release of resources in response to expected benefit. Moreover, because of its role in regulating corticostriatal plasticity in the indirect pathway, up- and down-regulation of D2R can regulate the stringency required for selectively releasing, via learning, indirect pathway inhibition of particular cortical afferents, potentially providing a mechanism by which the modulation of learning rate could also be linked to resource availability and cost control.

### Reduced D2R in Obesity: Reward or Energy Deficit?

The question of the relative contribution of D2R to regulating appetitive motivation vs. energy expenditure has been highlighted in recent years with the observation that D2R may be reduced in obesity (for review, Kravitz et al., 2016). Though accumulating studies have been inconsistent in this finding, the idea that D2R is reduced in obesity has fueled the notion that compulsive overeating can be construed as a food addiction, where reduced D2R signaling generates a “reward deficit” that drives behaviors, such as compulsive consumption of high energy, palatable foods (Volkow and Wise, 2005; Volkow et al., 2008; Kenny, 2011a,b; Kenny et al., 2013; Blum et al., 2014). This hypothesis centers around a role of D2R in regulating appetitive, incentive motivation, but entirely neglects any potential role of D2R in regulating energy expenditure and how that may contribute to obesity (Beeler et al., 2012c; Kravitz et al., 2016). In a recent study using D2R knockdown mice (D2KD; Beeler et al., 2016), we demonstrate that these mice show a pronounced reduction in behavioral energy expenditure, including homecage activity, open-field and wheel running, but no increase in appetitive motivation or consumption. When challenged on a high fat diet (HFD), the D2KD mice did not gain more weight than wild-type controls (slightly less, in fact) nor consume more of the palatable HFD. Provision of running wheels conferred substantial protection against dietary induced obesity in wild-type mice, but not in D2KD, who exhibited dramatically less running activity. This extended to protection against glucose dysregulation where wild-type with running wheels exhibited improved glucose clearance, while running wheels had no effect on clearance in D2KD. We further tested the mice in a concurrent choice paradigm to assess their willingness to work for preferred sucrose pellets. Again, the D2KD exhibited no evidence of increased appetitive motivation and did not work more for sucrose. Surprisingly, neither did they show decreased effort for sucrose pellets, contrary to our hypothesis. This could arise because the cost and amount consumed were low and not affected by reduced D2R signaling (i.e., low ratio strain) and/or because the reduced autoreceptor increased DA signaling that, via D1R activation in the direct pathway, effectively compensated by increasing facilitation for specific rewards. Interestingly, the D2KD mice with wheels exhibited a trend toward reduced breakpoint and greater thrift (Beeler et al., 2016, Figure 5), suggesting the possibility that the constraints on energy expenditure may be more apparent as options for behavioral activity increase. This work used a global D2R knockdown. As D2R is ubiquitously expressed, this limits the ability to attribute this regulation of energy expenditure specifically to striatal D2R on iMSNs. Friend et al. (2016) subsequently published an elegant study using selective deletion of D2R in iMSNs and obtained the same results—dramatically reduced activity but no increase in consumption, specifically confirming the importance of postsynaptic D2Rs on striatal iMSNs in regulating energy expenditure. These data suggest that while manipulations of D2R may affect appetitive behavior observed with pharmacological manipulations, this is likely to arise from a fundamental role of D2R in gating behavioral energy expenditure, which we interpret as encoding the value of resources to be expended, effectively mediating, or gating, cost considerations and implementing affordability.

One of the challenges of studying D2R is that it is ubiquitously expressed, making targeted manipulations and isolation of specific populations of D2R challenging. Even within the striatum, D2R is expressed on iMSNs, DA terminals (autoreceptors), cholinergic interneurons, afferent glutamatergic terminals and in a subset of direct pathway MSNs as D1-D2 heteromers. Though careful dissection of the function of these different populations of D2R largely remains to be tackled, the question moving forward is whether these different populations can, in a coordinated fashion, subserve different aspects of a single function—energy management, for example—and whether there is an coordination in their up- and down-regulation across different populations. For example, D2R expressed on incoming glutamatergic terminals has been proposed to act as a high pass filter, effectively filtering corticostriatal transmission selecting stronger afferent activity (Cepeda et al., 2001; Bamford et al., 2004; Centonze et al., 2004). Increases or decreases in presynaptic D2R expressed on glutamate afferents could increase or decrease the threshold determining what level of cortical activity is allowed through the high pass filter, potentially implementing a stringency for behavioral activation. A higher stringency for neurotransmission would increase the impact of prior learning in determining what cortical activity is and is not transmitted.

Regulation of energy expenditure by different striatal D2R populations might be more broadly observed in its role in regulating the DA system itself. Aside from D2R’s well-known role as autoreceptor (Ford, 2014), where it can alter DA signaling, for example enhancing sensitivity to amphetamine (Bello et al., 2011), it exerts control over DA signaling in numerous other ways. D2R is known to regulate DA reuptake and the DAT (Rougé-Pont et al., 2002; Bolan et al., 2007; Benoit-Marand et al., 2011; Owens et al., 2012) with increased D2R signaling increasing DAT function, altering the duration and summation of phasic increases in DA (Garris et al., 1994; Gonon, 1997). Chronic D2R activation can inhibit synaptogenesis in DA neurons (Fasano et al., 2008), again serving as a check on DA. In addition to D2R autoreceptors expressed on DA cells and terminals, evidence suggest that D2R expressed on MSNs may also negatively regulate DA synthesis/release and DA cell activity (Anzalone et al., 2012; Krabbe et al., 2015). D2R expressed in D1-D2 heteromers can have a tonic inhibitory effect and can enhance GABA production in the VTA (Perreault et al., 2015; Shen et al., 2015; Vekshina et al., 2017). While postsynaptic D2R on iMSNs appear to permissively gate energy expenditure, these other effects on the DA system appear, like the autoreceptor, to serve to limit dopaminergic activation. A crucial question is the relative regulation of these populations of D2R. If D2R increases on iMSNs, is this accompanied by a complementary *decrease* in D2R in these other, negative feedback populations, generating overall greater permissiveness, or by an similar increase, limiting the extent to which greater permissiveness in iMSNs can be leveraged, implementing a homeostatic mechanism to prevent the system from spiraling out of control.

## Regulation of Dopamine by Circulating Energy Signals

That the DA system is modulated by circulating energy signals has been clearly demonstrated over the last two decades (Figlewicz and Sipols, 2010; Vucetic and Reyes, 2010; de Araujo et al., 2011, 2012; Figlewicz, 2015), including insulin (Liu and Borgland, 2015), leptin (Fernandes et al., 2013), ghrelin (Perello and Dickson, 2015; Wei et al., 2015) and others (for example, GLP-1, Alhadeff et al., 2012; Dickson et al., 2012; Egecioglu et al., 2013). Most of this work has been developed within the framework of DA mediating appetitive drive rather than energy expenditure, suggesting that increases in energy signals diminish DA, effectively signaling satiety and reducing DA mediated appetitive drive. However, the idea that signals such as insulin and leptin will have a simple, unidirectional effect is proving inadequate to capture the complexity of the relationship between these signals and the DA system (e.g., leptin, Leinninger et al., 2009; Opland et al., 2010; Ribeiro et al., 2011). Using insulin as an example (reviewed in Liu and Borgland, 2015), insulin can regulate DAT expression and function (Carvelli et al., 2002; Garcia et al., 2005; Speed et al., 2011; Mebel et al., 2012; Kleinridders et al., 2015), TH activity (Figlewicz et al., 1996, 1998; Li et al., 2009; Könner et al., 2011), suppress afferent input onto DA cells and facilitate synaptic LTD onto DA cells (Labouèbe et al., 2013; Liu et al., 2013) and increase basal firing rate (Könner et al., 2011). While there is evidence that insulin can reduce DA mediated appetitive drive and reward (Figlewicz et al., 2006, 2007; Mebel et al., 2012), recent work has shown that in a hungry state, insulin increases DA release via insulin receptor activation of cholinergic interneurons that, in turn, enhance release from DA terminals (Stouffer et al., 2015).

Here again, we focus on D2R and ask whether D2R interactions with circulating energy signals could comprise an assessment of available energy resources necessary to determine the value of energy expended and implement cost controls. There is considerable evidence that the DA D2R plays a critical role in energy regulation generally, including interdependent regulation with both leptin and insulin. For example, D2R can regulate and mediate/modulate leptin (Kok et al., 2006; Kim et al., 2010) and insulin signaling (Figlewicz et al., 1998; Beaulieu et al., 2007; García-Tornadú et al., 2010; DeFronzo, 2011; Heni et al., 2015). Conversely, evidence suggest that D2R function can be regulated by both leptin (Pfaffly et al., 2010) and insulin (Sevak et al., 2005; Dunn et al., 2012; Owens et al., 2012). These data suggest an intimate link between D2R and systems regulating energy homeostasis; however, these studies either do not isolate a specific D2R population or they specifically identify peripheral populations, such as D2R in pancreatic islet cells (Lopez Vicchi et al., 2016). Though this is suggestive that D2R may play a critical role in energy management (Baik et al., 1995; Rowlett et al., 1995; Kelly et al., 1998; Chausmer et al., 2002; Tanabe et al., 2004; Sevak et al., 2006; Klinker et al., 2013; Beeler et al., 2016; Friend et al., 2016), there is much less data on how *striatal* D2R may be regulated by circulating energy signals. One study (Pfaffly et al., 2010) has directly linked leptin to regulation of striatal D2, where leptin increases D2R binding, consistent with our proposal that increased energy availability should increase D2R expression to facilitate greater energy expenditure and utilization. Another study found a negative relationship between insulin sensitivity and D2R binding potential (Dunn et al., 2012), which suggests that increased insulin sensitivity (associated with diminished caloric intake) would decrease D2R binding, again consistent with our hypothesis where a shortage of energy would reduce energy expenditure. While these limited studies directly examine the link between circulating energy signals and D2R are consistent with our hypothesis, further, more direct investigation is needed.

### D2R and Energetic Surplus

Most of the relevant work relating the regulation of D2R with energy availability has been conducted in either dietary induced obesity paradigms or, the opposite, food restriction (FR). Initial evidence suggested that D2R is reduced in obesity (Volkow et al., 2001, 2008; van de Giessen et al., 2013; de Weijer et al., 2014; Kessler et al., 2014) or in response to HFD, even without obesity (van de Giessen et al., 2013), but several studies have failed to replicate this finding (Dunn et al., 2010; Caravaggio et al., 2015; Cosgrove et al., 2015; Tuominen et al., 2015) or found changes in D2R associated with obesity to vary by striatal region (Guo et al., 2014; Adams et al., 2015). Animal studies have more consistently linked reduced D2R to obesity (reviewed in Kravitz et al., 2016), but interpreting these as a response to energy availability is fraught as both DIO and HFD can induce insulin resistance, which would paradoxically signal energetic scarcity despite surplus. Notably, reduced DA function has been inconsistently observed in DIO (Liu and Borgland, 2015; Décarie-Spain et al., 2016), an effect that is likely related to the length of time the animals are on the diet (Cone et al., 2013), suggesting that long-term adaptations, such as emergent metabolic disorder and insulin resistance, may underlie these reductions in DA function. Only one study looked at the effect of HFD after a short-exposure and observed an *increase* in D2R (South and Huang, 2008), consistent with our hypothesis that increased energy should elevate D2R to facilitate behavioral utilization of available energy. However, Sharma and Fulton (2013) subsequently observed increased D2R following 12-weeks of HFD. Thanos et al. (2008) demonstrate that FR increases striatal D2R in *obese* rats, apparently in contradiction to our hypothesis; however, they also demonstrate elevated circulating insulin and leptin in the obese rats compared to food restricted, indicative of glucose dysregulation and insulin resistance (Morris et al., 2011; Speed et al., 2011; Mehran et al., 2012; Fordahl and Jones, 2017). In our view, the most reasonable interpretation of the Thanos data is that FR increased leptin and insulin sensitivity (Fordahl and Jones, 2017), normalizing those systems, allowing these to effectively signal availability of energy. In a more recent human study (Dunn et al., 2012), the authors found that BMI and leptin was *positively* correlated with D2R, consistent with our hypothesis. The authors speculate this increased D2R availability reflects reduced endogenous DA competing with the radioligand rather than increased D2R, though determining which interpretation is correct will require further data.

### D2R and Energetic Scarcity

The effects of FR on the DA system has been less extensively investigated, despite the fact that the vast majority of operant behavioral studies used to characterize how the DA system modulates responding to environmental contingencies employ substantial FR, typically to 85% of baseline body weight. In an equivalent human study, this would mean reducing the weight of a 170 pound man to 145 pounds before starting the study, which we might expect to have profound effects on the subject and the behavior being studied, especially when the study centers on their response to opportunities for food. In our hypothesis here, we propose that conditions of energetic scarcity would induce an overall decrease in DA function to conserve energy. On first principles, we would predict a decrease in striatal D2R as well, to increase the stringency on the inhibitory “gate” regulating energy expenditure, particularly at basal, tonic levels of extracellular DA. Carr and colleagues have elegantly investigated the effects of FR on the DA system for many years and have found, generally, that FR sensitizes the DA system (Carr, 2002; Carr et al., 2003), enhancing the effects of drugs of abuse (Carr, 2007; Liu et al., 2011; Zheng et al., 2012). These effects, however, are complex. For example, although they observe an FR-induced up-regulation in TH mRNA, functionally this appears to occur in the context of a down-regulation of DA production (Pan et al., 2006). The net result might be characterized as a *down*-regulated DA system that exhibits *increased sensitivity* when activated (Pan et al., 2006), such as by drugs of abuse. Conceptually, this is consistent with our hypothesis: in environments of scarcity, down-regulated DA would decrease generalized energy expenditure but increase its energizing response to potential reward opportunity. However, contrary to our expectation, they have found that D2R function is increased under FR (Carr et al., 2003; Collins et al., 2008; Thanos et al., 2008; but see Sevak et al., 2008). This may reflect a compensatory up-regulation to increase sensitivity to reduced DA that results in chronically reduced receptor activation.

As with DIO, studies of FR are also confounded with potential effects on insulin signaling, as caloric restriction increases insulin sensitivity (Dostálová et al., 2007; Larson-Meyer et al., 2006; Weiss et al., 2006; Schenk et al., 2008, 2011; Mercken et al., 2012; Perez-Hedo et al., 2014; Salvador-Adriano et al., 2014), potentially enhancing its effects on DA signaling. In addition, caloric restriction also induces HPA activation and a glucocorticoid stress response (Deroche et al., 1993; Tomiyama et al., 2010; Pasiakos et al., 2011; Guarnieri et al., 2012; Grayson et al., 2014), which also affects DA system function (Abercrombie et al., 1989; Piazza and Le Moal, 1998; Yadid et al., 2001; Saal et al., 2003; Krishnan et al., 2007; Anstrom et al., 2009; Daftary et al., 2009; Rasheed et al., 2010; Cabib and Puglisi-Allegra, 2012; Tye et al., 2012; Chaudhury et al., 2013; Chang and Grace, 2014; Friedman et al., 2014; Sinclair et al., 2014; Hollon et al., 2015; Mantsch et al., 2016). In particular, chronic stress appears to induce, similar to FR characterized above, a generalized reduction in DA function with an increased sensitivity to stimuli indicative of reward (Mantsch et al., 2014; McReynolds et al., 2014; Belujon and Grace, 2015). Of course, adaptations in both insulin and stress signaling may represent primary mechanisms by which the DA system is adapted to conditions of energetic scarcity. Consistent with our hypothesis, stress has generally been shown to down-regulate D2R (Papp et al., 1994; Dziedzicka-Wasylewska et al., 1997; Gershon et al., 2007; Azzinnari et al., 2014), though this may be adaptive or maladaptive depending upon timing (Żurawek et al., 2013).

While FR certainly represents environmental scarcity, the degree to which it is a *good model* for environmental scarcity more broadly is questionable in that it: (a) represents an extreme, likely activating starvation responses (Duclos et al., 2013); and (b) the animal has no control over the starvation. That is, in a natural environment where an animal loses 15% of its body weight in a couple of days, this might reasonably trigger a fairly dramatic response to expend whatever energy is necessary to change the circumstances or face near-certain death; in short, this would not be the time to conserve energy. This is very different from an animal in an environment where food is available, but scarce and/or costly, requiring careful expenditure of energy in order to adapt to scarce resources.

### Environmental Enrichment

Whether investigating dietary surplus, i.e., DIO, or FR (explicitly, or simply employed as a method to motivate behavior in tasks), studies are almost entirely conducted in standard rodent housing conditions, which is in effect an impoverished environment (Würbel, 2001). Much as FR may induce starvation-related pathophysiology, the impoverished environment of standard rodent housing may also induce pathophysiology, including effects on DA function. Bardo and colleagues have been studying the effects of enriched environment on DA (largely focusing on the PFC) and susceptibility to drugs of abuse for many years (Stairs and Bardo, 2009). In general, animals in an enriched environment are less susceptible to addictive, compulsive pursuit of drugs (e.g., cocaine) than non-enriched animals (e.g., Green et al., 2010), but as with FR and DIO, the effects on the DA system are complex. Evidence suggest that enriched environments may up-regulate DA, both decreasing DAT expression and increasing DA release (Zhu et al., 2005; Niu et al., 2007; Zakharova et al., 2009; Segovia et al., 2010); however, animals in enriched conditions also show reduced locomotor activity in the open field (Bowling et al., 1993; Bowling and Bardo, 1994; Bardo et al., 1995; Green et al., 2010). Interestingly, animals in an enriched environment show a greater response to the acute locomotor effects of psychostimulants, consistent with upregulated DAT, but do not exhibit sensitization (Bowling et al., 1993; Bowling and Bardo, 1994; Bardo et al., 1995) and are less susceptible to addiction-like behaviors (Green et al., 2010). We could find no studies that directly examined D2R expression in response to environmental enrichment.

Like obesity and FR, the concept of environmental enrichment is fraught with difficulties. Enrichment has been defined and deployed in many ways and can include factors such as amount of space allotted to animals, number of conspecifics the animal is housed with (social), the provision of novel objects (toys, tunnels, changed regularly), the provision of a running wheel, or cognitive challenge (e.g., running wheels with missing rungs). One of the enduring difficulties in enrichment studies is determining the relative contribution of different aspects of enrichment to whatever subsequent behavioral or physiological differences may be observed (Simpson and Kelly, 2011; Grégoire et al., 2014). For example, given access to running wheels, rodents will generally run almost obsessively, dramatically increasing energy expenditure with a panoply of effects, including changes in glucose regulation (Hansen et al., 1998; Borghouts and Keizer, 2000), insulin receptor sensitivity (Ropelle et al., 2006; Bradley et al., 2008; Patterson et al., 2009; Krawczewski Carhuatanta et al., 2011; van Praag et al., 2014), glucocorticoid signaling (Droste et al., 2003, 2007; Nakajima et al., 2010; Clark et al., 2015; Chen et al., 2017) and the DA system (Gilliam et al., 1984; MacRae et al., 1987; Hattori et al., 1994; Fisher et al., 2004, 2013; Petzinger et al., 2007; Vučković et al., 2010; Eddy et al., 2014; Clark et al., 2015; Dang et al., 2017). Thus, in enriched environments with running wheels, observed effects could arise from environmental enrichment or from increased voluntary exercise and energy expenditure.

When asking, as we do here, how prevailing economic conditions—abundance or scarcity of resources—may alter DA mediated decision-making, we need to distinguish between an “enriched” environment in the sense of greater environmental complexity and a “rich environment” in terms of resource abundance. The hypothesis here centers on the latter, how DA adapts behavior to an *economic climate* of scarcity vs. plenty. In this sense, greater environmental complexity is most interesting when that complexity is related to opportunities to obtain and utilize resources, as in foraging paradigms discussed below. While studies of environmental enrichment have provided important insights, the question is whether this speaks more to the enrichment or to correction of the impoverished conditions of standard laboratory housing; that is, is “enrichment” enriched or simply *less impoverished*. The crucial question may lie in how different constellations of environmental characteristics, together with an environmental economy, induce a demand for adaptive behavior and how, in response, neural mechanisms and pathways might be modified to generate environment- and economy-specific adaptation.

## Integrative Environmental Complexity: Foraging Paradigms

As data accumulate and increasingly more sophisticated methods become available, including targeted genetic tools, opto- and pharmaco- genetic methods, the need for a larger conceptual framework on which to assimilate disparate but related information grows. While the work of Carr and colleagues on FR has its own purpose (addiction), as does the work of Bardo and colleagues with enriched environment (also addiction), as well as the many investigators that study feeding and obesity, all of these can be seen as different windows onto a larger system of organismal adaptation to the environment. A larger conceptual framework allows the relationships between disparate observations to be assimilated into a richer understanding.

We are not, of course, bereft of larger conceptual frameworks. Some view behavior as arising primarily from machine-like regulatory mechanisms (physiological, homeostatic), others as learned associations that generate behavioral responses to stimuli, essentially learned reflexes, still others see behavior as a computational problem. A larger conceptual framework serves not only to guide interpretation of data, but equally the formulation of questions and the design of experiments. Here we advocate for a neuroeconomic perspective: that behavior is a series of transactions with the environment in which the organism must optimize its return on the energy and resources (time, attention, memory) it expends. This decidedly does *not*, in our view, mean maximizing reward, but rather balancing the expenditure of resource assets to obtain additional resources and maintain an optimal state of “wealth” given specific environmental conditions, or economic climate in which the animal finds itself.

In order to see how different interlocking pieces of the puzzle fit together, there is a need for more naturalistic, more complex behavioral paradigms that offer the animals meaningful choices in response to environmental constraints and opportunities. Building on elegant work in behavioral ethology in the 70s and 80s, there has been a growing re-emergence of interest in foraging paradigms (Pearson et al., 2014; Calhoun and Hayden, 2015; Constantino and Daw, 2015). Broadly, in the sense intended here, foraging paradigms are those behavioral tasks in which the animal’s choices substantially alter the subjective, aggregate characteristics of the environment. As a rudimentary example, in our homecage progressive ratio, the average size of meals an animal chooses to eat determines the overall, average cost of food, as illustrated by hyperdopaminergic mice in this paradigm, who work twice as much for the same amount of food. In this example, the *average* cost of food is not fixed in the environment but arises as a consequence of the animal’s choices; the hyperdopaminergic mice experience a more costly environment. While the more common question in such paradigms is how alterations in particular neural substrates alter behavior, an equally important but less studied question is how different environmental conditions alter the neural substrates that mediate choice, which can in turn alter the subjective experience of the environment. A review of foraging or semi-naturalistic paradigms is beyond the scope of this review (see Pearson et al., 2014; Calhoun and Hayden, 2015); however, we wish to highlight that as our knowledge accumulates on the neural substrates regulating behavior, more naturalistic paradigms that allow us to observe how atomistic components of behavior are integrated in response to complex environments will become increasingly necessary.

## Normative Models of Neurophysiological Substrate Adaptations

Richer, more complex behavioral paradigms require richer interpretive models. Theoretical, computational neuroscience is in renaissance, providing elegant models of behavior and neural function increasingly linked empirically to underlying neural substrates. Within the DA field, temporal difference learning models (Schultz et al., 1997; Sutton and Barto, 1998) have changed the theoretical landscape, providing rigorous formal theories for understanding DA function and its mediation of choice behavior. In more complex paradigms, formal modeling is often necessary to understand data that cannot be boiled down to a simple metric, such as breakpoint in progressive ratio. While such models are typically anchored in either behavioral or physiological data, they have not generally been integrated with what might be considered non-normative, non-computational physiological processes linked to more historical perspectives, such as homeostatic mechanisms or, as discussed here, changes in the computational substrate itself. That is, how up- or down-regulation of DAT, release probability, or receptors might change decisions arising from temporal difference computational algorithms has largely not been addressed, with notable exceptions. Keramati and Gutkin (2014) developed a model on how physiological “drive” mechanisms could be instantiated through TD algorithms to maintain homeostasis. Frank et al. (2009), Collins and Frank (2014) and Cox et al. (2015) are developing models in which DA value and error signals are parsed into separate channels signaling through D1R and D2R receptors, linking these with positive and negative prediction errors, respectively. Like behavior paradigms, computational models are simplifications; such simplifications often include ignoring “analog” adaptations such as up-regulating a gene, altering release probability and so on. An important task for future theoretical modeling might be to tame these messy biological adaptations by incorporating them into normative models; that is, the up-regulation of DAT changes DA signaling, but under what conditions should DAT be up- or down-regulated in order to achieve optimal decision-making and behavior? There is a broad vista open for incorporating these permutations in biological substrate as functional, parameterized components of formal models. While some changes in biological substrates may simply alter existing components of a model, such as inverse temperature (e.g., Beeler et al., 2010), learning rate (Frank et al., 2009) or delay discounting, others may be less definable within the current terms of the models, requiring elaboration and development, such as Frank et al. (2009) separating positive and negative signals based on D1R and D2R transmission. Our hypothesis, outlined here, suggest one aspect not incorporated into current models: the question of affordability—how the availability or wealth of resources necessary to pursue reward—are calculated into cost-benefit decisions.

## Future Directions

Construing the DA system as a central regulator of resource expenditure, most fundamentally energy, provides a useful framework for integrating many observations and apparently disparate functions of the midbrain DA system. DA is often associated with compulsive behavioral disorders, such as addiction and obesity. Elsewhere, we have proposed that DA might be better construed as mediating behavioral flexibility rather than simply driving behavior toward reward (Beeler et al., 2014a,b). A critical aspect of flexible behavioral adaptation is “living within one’s means”; that is, adapting choices and expenditure of resources to resource wealth. One of the most interesting aspects of this perspective is that behavioral choices can shape individual, subjective experience of an environment as much as the actual characteristics of the environment itself (e.g., Wikenheiser et al., 2013). While psychology has long been interested in subjective experience and *perceived* environments (e.g., perceived stress, Gibson’s “affordances”), the notion that the computations an animal performs in assessing and interacting with its environment can substantively alter the experienced characteristics of that environment provides a window onto the idea of *experienced environment* that is concrete and can be formalized and studied in animal models (e.g., Wikenheiser et al., 2013). While foraging theory sought to understand how behavior is optimized (Charnov, 1976; Stephens and Krebs, 1986), and more recently the neural substrates that mediate such optimized behavior (Pearson et al., 2014; Calhoun and Hayden, 2015), an extension of this is to systematically characterize and formally describe suboptimal behavior—and its consequences—and the changes in neural substrates that produce it.

DA abnormalities have been implicated in numerous neuropsychiatric disorders, often framed in terms of reward processing. However, altered regulation of energy expenditure is a characteristic across many disorders, including depression, addiction, schizophrenia and attention-deficit hyperactivity disorder. An essential aspect of these disorders might be dysfunction in how the brain allocates energy and resources in economic decision-making. There is an increasing call to start to rethink psychiatric disorders in theoretical, computational terms, as computational systems gone awry (Sharp et al., 2012; Culbreth et al., 2016; Gillan et al., 2016; Huys et al., 2016). In doing so, we suggest that the crucial evolutionary computational problem is adapting energy expenditure to the environmental economy—to live on a budget—in order to maximize probability of survival. One approach is to link formally described suboptimal decision-making, as noted above, with observable behavioral characteristics under an umbrella of “scarcity (or surplus) phenotype”. This characterization can be further elaborated by considering that suboptimal behavior could arise from different origins: *real* scarcity in the environment (e.g., poverty), *false* scarcity arising from pathophysiology in computational substrates (e.g., insulin resistance) or *induced* or experienced scarcity arising from suboptimal, maladaptive choices.

In an interesting study, Mani et al. (2013) showed that cycles of economic plenty and scarcity altered cognitive function in farmers. Both human and animal studies show that animals can adapt decision-making strategies to environmental conditions (Kolling et al., 2014; Kwak et al., 2014). Intuitively, it is not difficult to imagine that our environmental conditions, above and beyond stress, could alter how our brains make computations that determine our response to the world around us—arising not just from different inputs to these computations, but from alterations in the computational substrates themselves. Such neural adaptations to environmental and economic conditions are surprisingly understudied, though presumably they underlie a great deal of behavioral variability. Given its centrality to decision-making, motivated behavior, reinforcement learning and behavioral energy expenditure—as well as well-developed formal computational models—DA is an ideal target to begin to ask and investigate the fundamental question: how does our brain adapt neural processing and decision-making to our economic environment? In our view, the evolutionarily ancient neuromodulator DA (Vidal-Gadea and Pierce-Shimomura, 2012), with its widespread modulatory effects on the mammalian brain (Decot et al., 2017), is situated to be a central substrate mediating this economic adaptation, implementing a neurobehavioral organismal resource budget and incorporating affordability into decision-making.

## Author Contributions

JAB: conceived and wrote the hypothesis and manuscript. DM: contributed to development of the hypothesis and wrote the manuscript.

## Conflict of Interest Statement

The authors declare that the research was conducted in the absence of any commercial or financial relationships that could be construed as a potential conflict of interest.
